# Quality of postoperative pain management in Ethiopia: A prospective longitudinal study

**DOI:** 10.1371/journal.pone.0215563

**Published:** 2019-05-01

**Authors:** Million Tesfaye Eshete, Petra I. Baeumler, Matthias Siebeck, Markos Tesfaye, Abraham Haileamlak, Girma G. Michael, Yemane Ayele, Dominik Irnich

**Affiliations:** 1 Department of Anesthesiology, Institute of Health, Faculty of Medicine, Jimma University, Jimma, Ethiopia; 2 CIHLMU Center for International Health, Medical Center of the University of Munich (LMU), Munich, Germany; 3 Multidisciplinary Pain Center, Department of Anesthesiology, University Hospital, Ludwig Maximilians University (LMU), Munich, Germany; 4 Department of General, Visceral und Transplantation Surgery, Medical Center of the University of Munich (LMU), Munich, Germany; 5 Department of Psychiatry, St. Paul's Hospital Millennium Medical College, Addis Ababa, Ethiopia; 6 Department of Pediatrics and Child Health, Institute Of Health, Faculty of Medicine, Jimma University, Jimma, Ethiopia; Cleveland Clinic, UNITED STATES

## Abstract

**Background:**

The annual number of surgical operations performed is increasing throughout the world. With this rise in the number of surgeries performed, so too, the challenge of effectively managing postoperative pain. In Africa, there are scanty data available that make use of multi-center data to characterize the quality of postoperative pain management. In this study using a longitudinal data, we have attempted to characterize the quality of postoperative pain management; among patients scheduled for major elective orthopedic, gynecologic and general surgery.

**Methods:**

This prospective longitudinal study evaluated the quality of postoperative pain management in patients undergoing elective general, gynecologic, and orthopedic surgery. We quantified the prevalence of moderate to severe postoperative pain with the International Pain Outcome Questionnaire and the corresponding adequacy of treatment with the pain management index. At four time points after surgery, we estimated pain severity, its physical and emotional interference, and patient satisfaction.

**Results:**

Moderate to severe postoperative pain was present in 88.2% of patients, and pain was inadequately treated in 58.4% of these patients. Chronic pain (β = 0.346, 95% CI: 0.212, 0.480) predicted patients’ worst pain intensity. Gender was not associated with the worst pain intensity or percentage of time spent in severe pain. Patient’s pain intensity did not predicted the level of satisfaction.

**Conclusions:**

The prevalence of moderate to severe postoperative pain and its functional interference is high in Ethiopian patients. The treatment provided to patients is inadequate and not in line with international recommendations and standards.

## Introduction

In the year 2012 alone, 266.2 to 359.5 million operations were performed, representing a 38% increase over the previous eight years; low-income countries thereby had the most dramatic increase [1]. The rise in the number of operations is not without risk; for example, after analyzing 5000 patients a study acknowledged that 22% of chronic pain is caused by surgery [2]. Postoperative pain is still problematic, and up to 40% of patients have severe pain [3].

Untreated postoperative pain has dangerous consequences, ranging from prolonged duration of the hospital stay to more severe complications, such as chronic pain, atelectasis, respiratory infection, myocardial infarction [4, 5], and even death [6]. Several risk factors have been identified for severe postoperative pain, but epidemiological studies often have conflicting results about the relevance of such risk factors [7]. Furthermore, almost all investigations related to the topic were conducted in well-resourced settings. Because of obvious variances in health care across settings, it cannot be assumed that the same risk factors, trends, and magnitude of acute postoperative pain exist in settings with limited resources.

Developing countries tend to prioritize the eradication of poverty and hunger and reduction of maternal and child mortality and pay little attention to pain management [8]. Global health policy also turns a blind eye to the consequences of uncontrolled pain and the associated global burden [5]. Although pain management continues to be a problem in both developed [3] and developing countries [9], sadly the suffering from untreated pain is larger and more troublesome among the economically disadvantageous individuals [5]. Hence, this study is relevant to society because it gives a voice to the voiceless postoperative patients in low-resource settings. This applies especially to those from the least developed countries, such as Ethiopia, where there is still no scientific interest in the quality of postoperative pain treatment. For example, to our knowledge at the time of writing this report only one study has been conducted on the burden of postoperative pain in Ethiopia [10].

Therefore, this prospective longitudinal study from three tertiary care hospitals assessed the magnitude of and risk factors for postoperative pain. We also estimated key epidemiological profiles that characterize the quality of postoperative pain therapy in Ethiopia on the basis of data obtained from three large teaching and referral hospitals.

## Methods

### Design and setting

From September 11^th^ to December 17^th^ 2016, we conducted a longitudinal study of elective surgical patients to determine the quality of postoperative pain management in Ethiopia. The study involved repeated measures of patient-reported postoperative pain outcomes. Three state-owned teaching and referral hospitals were selected. Two of the chosen sites, Zewditu Memorial Hospital (ZMH) and Yekatit 12 Hospital Medical College (YK12HMC), are located in the capital, Addis Ababa. The city has approximately 3,197,000 inhabitants [11]. ZMH is one of the main tertiary referral hospitals and serves a population of over 600,000; it provides surgical, medical, and emergency care (inpatient and outpatient) and has an average of 180 beds. YK12HMC is a 340-bed referral hospital under the Addis Ababa City Government Health Bureau that provides services for about 4 million people from its catchment area, Arada sub-city and the neighboring sub-city and Oromia region. The third hospital is Jimma University Medical Center (JUMC), which is located 350 km south-west of the capital. It is the largest teaching and referral hospital in the southwestern part of the nation; with 634 beds, it provides services for a catchment population of about 15 million people.

### Ethics statement

The study was approved by the Jimma University Institutional Review Board, ref. no. RPGC/06/2016, and the Medical Ethics Committee of the Ludwig Maximillian University, Munich, Germany, ref. no. 17–224. All participating hospitals also granted permission for the study (ref. nos. ጤምድምማ/ 567/2008, ጤምድምማ/ 568/2008, ጤምድምማ/ 569/2008). The study was carried out in compliance with the Declaration of Helsinki. Patients were given comprehensive oral and written information on the purpose and procedures of the study. Before inclusion, participants provided written informed consent and were informed about their rights to refuse to participate or withdraw from the study at any time. They were also informed about the confidentiality of the information gathered during the study and that any personal information would be anonymized before the final analysis.

### Participants

The night before planned operations, we identified eligible patients on the surgical waiting list and approached them to explain the study objectives and measures. We recruited 356 consecutive patients. The inclusion criteria were as follows: adult surgical inpatients aged 18 years or older scheduled for general, orthopedic, or gynecologic surgery. The exclusion criteria were cognitive and mental disabilities (identified in patients’ clinical records); direct transfer to an intensive care unit; and emergency surgery, including cesarean section. In addition, those who did not stay in the hospital overnight after their surgical procedure were excluded.

### Outcome measures

Pain outcome variables after surgery were measured by the International Pain Outcome Questionnaire (IPOQ), which was originally developed from the American Pain Society Patient Outcome Questionnaire (APSPOQ) [12]. The IPOQ has been translated into 15 different languages and validated in 8 European countries and Israel [13]. It includes questions on pain severity, pain interference with physical function and emotions, side effects of pain treatment, and perception of care. Also, it can be used to gather information on the use of non-pharmacological methods for pain relief and the presence of preoperative chronic pain. IPOQ items are scored mostly on an 11-point numeric rating scale (NRS; scores 0–10), but the questionnaire also includes “yes” and “no” responses. Patients’ worst, least, and current pain intensity was measured as an NRS score from 0 = “no pain” to 10 = “worst pain possible.” The percentage of time the patient had spent in severe pain since surgery was measured from 0% = “never in severe pain” to 100% = “always in severe pain.” Pain interference was measured as functional disability due to pain (NRS score from 0 = “did not interfere” to 10 = “completely interfered”) and anxiousness and helplessness caused by pain (NRS score from 0 = “not at all” to 10 = “extremely”).

Adequacy of pain management was measured with the Pain Management Index (PMI). The index is calculated by first categorizing patients’ worst pain intensity into 0 (no pain), 1 (1–3: mild pain), 2 (4–6: moderate pain), and 3 (7–10: severe pain). The final score is then subtracted from the strength of analgesic prescribed: 0 (no analgesic drug), 1 (non-opioids), 2 (weak opioids), and 3 (strong opioids). The final score ranges from –3 to +3, and negative scores indicate inadequate treatment. Originally, this index was designed to assess the adequacy of cancer pain management; however, it has also been used in surgical patients [14–16]. Patient perception of care was measured as the degree of pain relief through pain treatment (NRS score from 0% = “no relief” to 100% = “complete relief”). Patients’ wishes for more analgesics were recorded as “yes” or “no.” Satisfaction with the results of pain treatment was measured as an NRS score from 0 = “extremely dissatisfied” to 10 = “extremely satisfied.” For this study, the original English version of the PMI was translated (forward and backward) into two local languages and pilot tested in five steps, as per international guidelines [17]. The final version was approved by an expert panel to ensure content and face validity.

### Measurements

At all three hospitals, outcome variables were measured 6, 12, 24, and 48 hours after surgery. Interviewers administered the IPOQ; such a method of administration is justified when patients are too ill, in too much pain, or—as was the case in our setting—unable to read or write. To avoid measurement bias, the nurses who collected data did not participate in treating the respective study participant at the time of data collection.

### Covariates

The study considered the following covariates: time (since surgery), patient’s age and sex, pre-existing chronic pain, and time of the operation. We also retrieved information on demographics, medical history, type of surgery, type of anesthesia, patient’s physical condition, and pain treatment from the medical records.

### Statistical analysis

We used generalized estimating equations (GEE) for the analysis because we wanted to model the change in outcome measures over time [18] and were interested in population-averaged effects rather than subject-specific effects [18]. Throughout the analysis, we used a manual stepwise backward elimination approach to select covariates that influenced the time course of the different outcome measures. We evaluated the best-fitting model and working correlation structure by quasi-likelihood under independence criteria (QIC) and corrected quasi-likelihood under independence criteria (QICu) and chose the best-fitting model with the lowest possible value [19]. QIC is the modification of the Akaike information criterion (AIC) for the GEE. Consequently, we used an exchangeable working correlation structure with Huber-White standard error estimates (robust standard error) for all GEE analyses [18].

The linear relationship between outcomes and time was analyzed by adding time squared to the GEE model. In case of a non-linear relationship, time was included in the model as a categorical variable. The GEE equation, which allows one to adjust for the dependency of observations within one subject, is as follows:

$$Y_{it=}\beta_{0}+\sum_{j=1}^{J}\beta_{1}{}_{j}X_{itj}+....+CORR_{it}+\varepsilon_{it}$$

in which Y_it_ is the observed outcome for the subject i at time t, β0 is the intercept, X_ijt_ is the covariate j for the subject i at time t, β_1j_ is the regression coefficient for covariate j, J is the number of covariates, CORR_it_ is the working correlation structure, and εit is the “error” for subject i at time t. A p value of 5% was considered significant, and all analyses were performed with Stata version 13.0 (StataCorp., Texas, USA). Graphs were produced using the ggplot2 package [20] and the R software [21].

## Results

### Demographic and clinical information

All eligible patients agreed to participate in the study. There were slightly more women than men (51.1%; mean [SD] age of the women: 35.4 [0.9] years; mean [SD] age of the men: 44.5 [0.67] years). The majority of participants were Ethiopian Orthodox Tewahedo Christians, and Oromo was the dominant ethnic group. According to the medical records, almost all patients had an American Society of Anesthesiologists Physical Status Classification 1 (ASA PS 1). The median (IQR) duration of the surgery was 1.3 (1–2) hours. Most patients underwent general anesthesia; one third spinal anesthesia; and only five patients, ketamine anesthesia. The predominant type of surgery was cholecystectomy, followed by thyroidectomy and prostatectomy. Details of the social and clinical demographics are provided in Table 1. Tramadol (93%) took the greater share of the analgesics prescribed, followed by diclofenac (7%). Of these about 53.7% of the analgesics were prescribed by the surgical resident, 43.3% by the Surgeon, 2.8% by the medical intern and about 0.3% by the anesthesiologist.

**Table 1:** Demographic and clinical characteristics*.

Age in years, mean (SD)	39.9 (16.3)	
Duration of surgery in hours, median (IQR)	1.5 (0.73)	
	n	%
Women	182	51
Physical status classification		
ASA PS 1	347	98
ASA PS 2	9	2.5
Educational status		
Illiterate	133	38
Elementary school	107	48
High school	54	24
Certificate	25	11
Diploma	26	12
Degree and above	11	4.9
Religion		
Orthodox Christian	212	60
Muslim	126	35
Protestant	18	5.1
Marital status		
Married	253	71
Single	82	23
Divorced/widowed	21	5.9
Ethnic group		
Amhara	142	40
Oromo	147	41
Others*	67	19
Type of anesthesia		
General Anesthesia	247	69.4
Spinal Anesthesia	104	29.2
Ketamine Anesthesia	5	1.4
Type of surgery		
General surgery	109	30.6
Orthopedic surgery	197	55.3
Gynecologic surgery	50	14.0

*Tigre, Wolayta, Gurage, Kafa, Silte.

### Adequacy of pain management and perception of care

When we used the patients’ worst pain intensity as a reference, the time course of the PMI scores indicated that 58.4% of patients were inadequately treated during the first 6 postoperative hours. Moderate to severe postoperative pain was reported by 88% of patients at 6 hours after surgery and by 40% of patients at 48 hours after surgery. When asked whether they needed more analgesics than prescribed, 57% of the patients replied “yes” at 6 hours after surgery (95% CI: 52.1%, 62.4%) and 55% did so at 12 hours after surgery (95% CI: 49.5%, 59.9%). This figure dropped to 37% (95% CI: 31.9%, 42.0%) at 48 hours after surgery. None of the patients in our sample received any information about options for pain treatment. Patient pain was treated predominantly with tramadol (92.9%) and sometimes with diclofenac (7.0%). The most prevalent non-pharmacological method of pain management was talking to friends or relatives; this method was used by 88.3% (95% CI: 82.5%, 92.4%) of the patients at 6 hours after surgery; by 90.6% (95% CI: 85.2%, 94.2%), at 12 hours after surgery; by 90.1% (95% CI: 84.5%, 93.8%), at 24 hours after surgery; and by 94.7% (95% CI: 90.1%, 97.3%), at 48 hours after surgery.

### Pain intensity

#### A. Worst pain intensity

**Fig 1:**
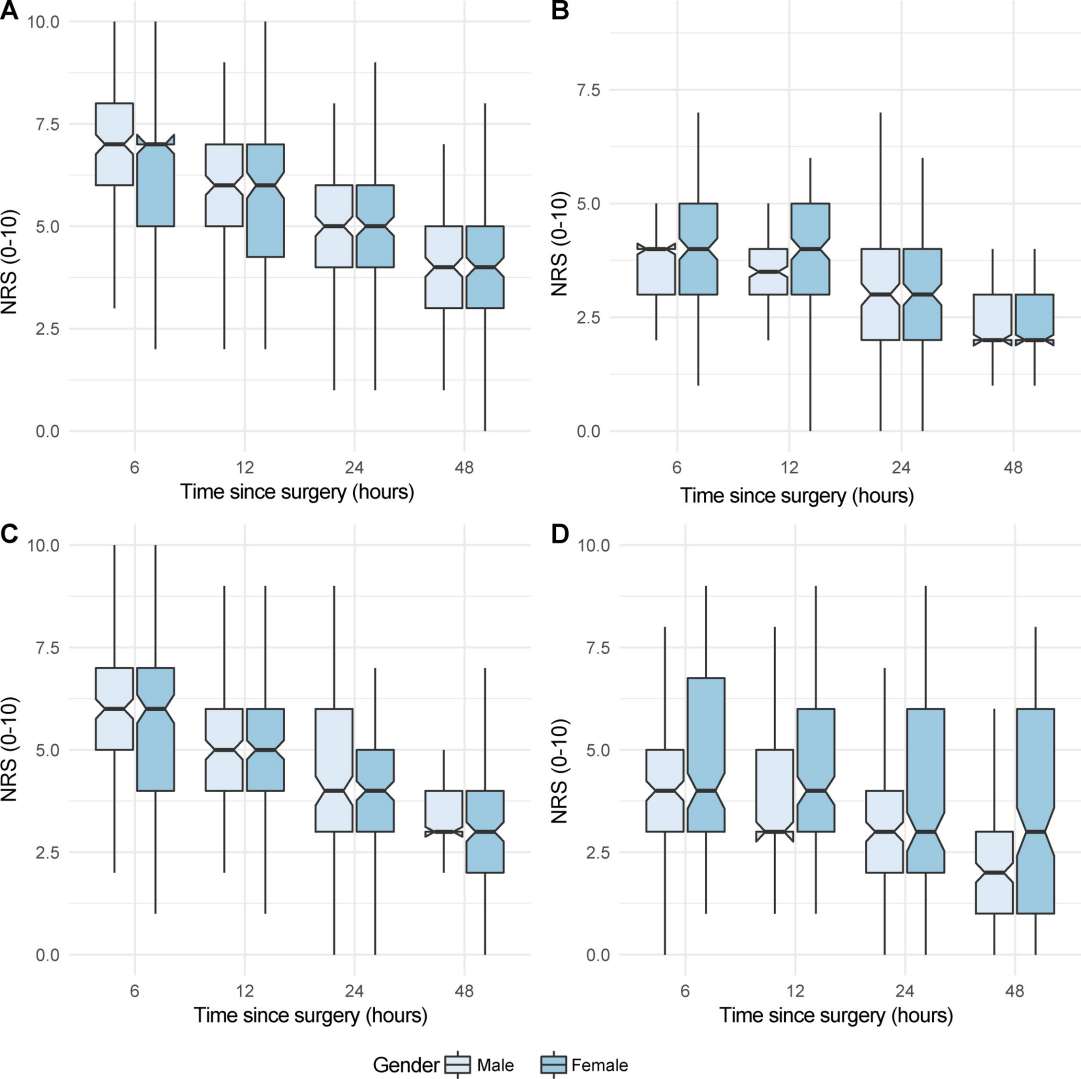
Numeric rating scale (NRS) scores for pain intensity measures in male and female patients. (A) Worst pain; (B) Least pain; (C) Current pain; and (D) Time spent in severe pain.

The worst pain intensity ratings had mean (SD) NRS scores of 6.5 (1.63) at 6 hours after surgery, 5.7 (1.6) at 12 hours, 4.9 (1.6) at 24 hours, and 4.2 (1.4) at 48 hours. The patients’ current and least pain intensity also declined over time but did not differ between the sexes. However, it is noteworthy that 88% of the participants had moderate to severe pain during the first 6 hours after surgery. Even at the subsequent assessments, the prevalence of moderate to severe postoperative pain was still high: 77% at 12 hours, 63% at 24 hours, and 40% at 48 hours after surgery. The median value for all pain intensity measures across time are shown in Fig 1.

The QIC statistic for GEE model selection suggested age, sex, educational status, type of anesthesia, type of surgery, chronic pain severity, and time since surgery as covariates for the final model. The patient’s worst pain intensity rating was affected by time since surgery, age, chronic pain severity, and educational status. In comparison to the pain at 6 hours after surgery, the worst pain intensity was significantly lower at each subsequent measurement time point: 12 hours (β = -0.66, 95% CI = -0.946, -0.375), 24 hours (β = -1.49, 95% CI: -1.758, -1.228), and 48 hours (β = -1.988, 95% CI: -2.315, -1.661). With increasing age, the worst pain intensity decreased (β = -0.018, 95% CI: -0.032, -0.004). Larger NRS scores of preoperative chronic pain were associated with a higher worst pain rating after surgery (β = 0.346, 95% CI: 0.212, 0.480). Illiterate patients had higher worst pain intensity scores (β = 0.552, 95% CI: 0.1562, 0.94731) than those with formal education. Sex, type of anesthesia, type of surgery, duration of surgery, and physical status did not affect the patients’ worst pain experience (see Table 2).

**Table 2:** Multivariate model predicting pain intensity over time*.

Predictors of worst pain	Coefficient (95% CI) ^†^	P Value
Age	-0.018 (-0.032, -0.004)	0.014
Educational status		
Illiterate	0.552 (0.156, 0.947)	0.006
Chronic pain severity	0.346 (0.212, 0.480)	<0.01
Time since surgery		
12 h	-0.660 (-0.946, -0.375)	<0.01
24 h	-1.493 (-1.758, -1.228)	<0.01
48 h	-1.988 (-2.315, -1.661)	<0.01
**Predictors of time Spent in severe pain**		
Marital status		
Single	0.752 (0.012, 1.492)	0.046
Divorced/widowed	0.453 (-0.986, 1.892)	0.537
Ethnic group		
Oromo	-0.992 (-1.714, -0.270)	0.007
Others**	-0.122(-0.902, 0.658)	0.759
Religion		
Muslim	-1.338 (-2.017, -0.658)	<0.01
Protestant	-2.056(-2.781, -1.332)	<0.01
Types of Anesthesia		
Ketamine anesthesia	1.436 (0.195, 2.677)	0.023
Duration of surgery	0.968 (0.568, 1.369)	<0.01
Chronic pain severity	0.239 (0.041, 0.436)	0.018
Time since surgery		
12 h	-0.123 (-0.409, 0.163)	0.401
24 h	-0.760 (-1.056, -0.464)	<0.01
48 h	-1.127(-1.414, -0.839)	<0.01

Abbreviations: CI, confidence interval.

*Variables tested that did not predict worst pain included gender, physical status, type of anesthesia and type of surgery. Whereas variables that did not predict time spent in severe pain are age, gender, educational status, physical status and type of surgery. Because of non-linearity, time was entered into the model as a factor variable in the final model.

†For predictors with only main effects, the coefficient inference from the reference group (e.g., the reference group for marital status is married, for ethnic group is Amhara, for religion is Orthodox, for type of anesthesia is general anesthesia, for type of surgery is general surgery and for time since surgery is 6 hours).

**Tigre, Wolayta, Gurage, Kafa, Silte.

#### B. Time spent in severe pain

The NRS scores for the mean (SD) time spent in severe pain were 4.4 (2.0) at 6 hours, 4.2 (1.98) at 12 hours, 3.7 (1.99) at 24 hours, and 3.1 (2.3) at 48 hours. In addition to the predictors of worst pain, the QIC statistic informed the inclusion of ethnic group, religion, marital status, and duration of surgery. Single participants reported higher percentages of time spent in severe pain than married participants (β = 0.752, 95% CI: 0.012, 1.492), and Muslim and Protestant patients reported less time spent in pain than Orthodox Christian patients (β = -1.338, 95% CI: -2.017, -0.658 and β = 2.056, 95% CI: 2.781, 1.332, respectively). The longer the duration of surgery in hours, the higher the rating for time spent in severe pain (β = 0.968, 95% CI: 0.568, 1.369). NRS scores of preoperative chronic pain also predicted how much time patients spent in severe pain (β = 0.239, 95% CI: 0.041, 0.436). The NRS scores of time spent in severe pain did not differ significantly at 12 hours after the surgery compared with at 6 hours; however, time spent in pain subsequently decreased significantly at 24 hours (β = -0.76, 95% CI: -1.056, -0.464) and 48 hours (β = -1.13, 95% CI: -1.414, -0.839). Age, sex, type of surgery, type of anesthesia, educational status, and ASA PS classification were not associated with the time spent in severe pain (see Table 2).

### Pain interference

**Fig 2:**
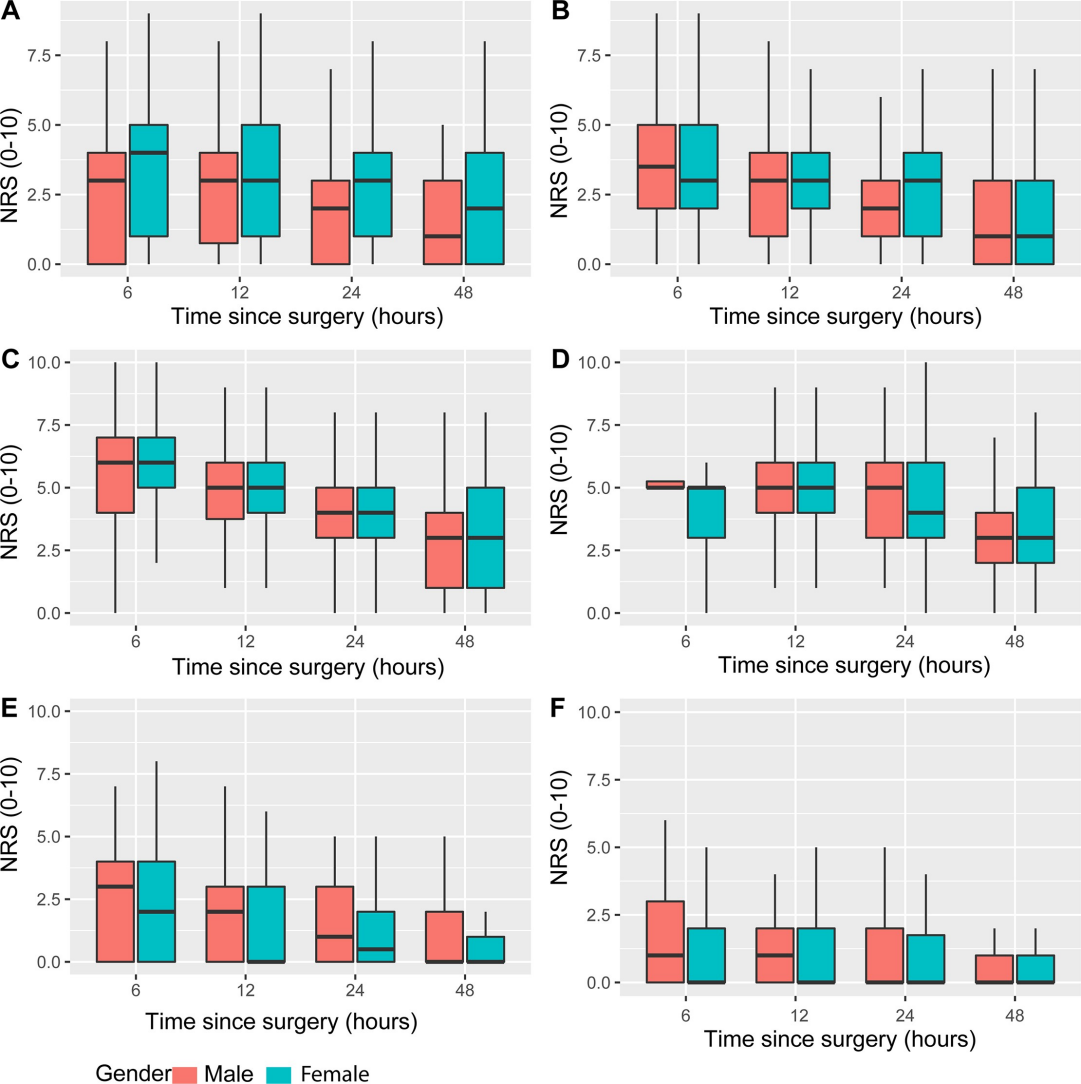
Numeric rating scale (NRS) scores for pain interference. Pain interference with function (A) breathing and coughing; (B) sleeping; (C) movement; and (D) activities in bed; and causing (E) anxiousness; (F) helplessness in male and female patients after surgery.

The trend of pain interference with function (i.e., breathing and coughing, sleeping, movement, and activities in bed) and emotions (anxiousness and helplessness) over time is depicted in Fig 2.

#### A. Interference with movement

Pain interference with movement was moderate, with mean (SD) NRS scores of 4.5 (1.9), 4.97 (1.7), 4.54 (1.9), and 3.30 (1.92) at 6, 12, 24, and 48 hours after surgery, respectively. ASA PS 2 patients reported greater interference (β = 0.942, 95% CI: 0.250, 1.633) than ASA PS 1 patients. Compared with patients who underwent general anesthesia with endotracheal intubation, those who underwent spinal anesthesia had higher scores of pain interference with movement (β = 0.726, 95% CI: 0.256, 1.23). Patients’ ratings of the worst (β = 0.363, 95% CI: 0.225, 0.496) and current (β = 0.373, 95% CI: 0.235, 0.511) pain intensity also affected their mobility. When the score for perceived pain relief increased, pain interference with movement decreased significantly (β = -0.027, 95% CI: -0.040, -0.014). Interference of pain with movement was also affected by the level of education: illiterate patients reported more interference than literate ones (β = 0.503, 95% CI: 0.028, 0.978). None of the variables time since surgery, ethnic group, time spent in severe pain, level of education, religion, chronic pain severity, and type of surgery had an effect (see Table 3).

**Table 3:** Multivariate model predicting pain interference over time*.

Predictor of pain interference with movement	Coefficient (95% CI)^†^	P value
Educational status		
Illiterate	0.478 (0.004,0.952)	0.048
Marital status		
Single	-0.670 (-1.316,-0.023)	0.042
Widowed/divorced	-0.338 (-1.020,0.343)	0.330
Physical Status		
ASA PS 2	0.922 (0.231,1.613)	0.009
Type of anesthesia		
Spinal anesthesia	0.706 (0.246,1.166)	0.003
Duration of surgery	0.179 (-0.155,0.512)	0.295
Chronic pain severity	0.161 (0.057,0.265)	0.002
Pain intensity		
Worst pain	0.366 (0.225,0.507)	<0.01
Current pain	0.390 (0.230,0.551)	<0.01
Time in pain	0.008 (-0.001,0.018)	0.093
Perceived care		
Relief received	-0.027 (-0.041,-0.013)	<0.01
**Predictors of pain interference with activities in bed**		
Time since surgery, h	-0.021 (-0.027, -0.015)	<0.01
Worst pain	0.319 (0.225, 0.413)	<0.01
Current pain	0.282 (0.174, 0.390)	<0.01
Time in severe pain	0.021 (0.015, 0.027)	<0.01
**Predictors of pain interference with sleep**		
Worst pain	0.352 (0.230, 0.475)	0.001
Current pain	0.302 (0.163, 0.440)	0.001
Time in severe pain	0.021 (0.007, 0.034)	0.003
Perceived relief	-0.022 (-0.044, -0.001)	0.044
**Predictors of pain interference with breathing and coughing**		
Physical status		
ASA PS 2	0.671 (0.022, 1.321)	0.043
Type of anesthesia		
Spinal anesthesia	-1.222 (-1.879, -0.565)	<0.01
Ketamine anesthesia	-0.194 (-1.292, 0.904)	0.729
Chronic pain severity	0.253 (0.100, 0.407)	0.001
Type of surgery		
Gynecologic surgery	0.099 (-0.476, 0.674)	0.736
Orthopedic surgery	-1.235 (-2.135, -0.335)	0.007
Time since surgery	-0.015 (-0.026, -0.004)	0.006
Pain intensity		
Worst pain	0.199 (0.051, 0.347)	0.008
Current pain	0.268 (0.145, 0.391)	<0.01
Perception of care		
Relief received	-0.020 (-0.037, -0.004)	0.016
**Predictors of pain causing the feeling of anxiousness**		
Marital status		
Single	-0.957 (-1.649,-0.265)	0.007
Widowed/divorced	0.549 (-1.183,2.280)	0.535
Type of anesthesia		
Spinal anesthesia	0.087 (-0.658,0.831)	0.820
Ketamine anesthesia	-1.178 (-1.981,-0.376)	0.004
Duration of surgery	-0.117 (-0.609,0.375)	0.641
Chronic pain severity	0.179 (0.005,0.352)	0.044
Type of surgery		
Gynecologic surgery	-1.002 (-1.685,-0.319)	0.004
Orthopedic surgery	0.161 (-0.835,1.157)	0.752
Time since surgery	-0.004 (-0.014,0.005)	0.379
Pain intensity		
Worst pain	0.308 (0.179,0.437)	<0.01
Current pain	0.253 (0.131,0.375)	<0.01
**Predictors of pain causing the feeling of helplessness**		
Marital status		
Single	0.727 (-1.408, -0.046)	0.036
Widowed/divorced	0.799 (-1.044, 2.643)	0.395
Religion		
Muslim	0.418 (0.003, 0.833)	0.049
Protestant	-0.273 (-0.817, 0.272)	0.326
Type of anesthesia		
Spinal anesthesia	0.280 (-0.440, 0.999)	0.446
Ketamine anesthesia	-1.494 (-2.305, -0.684)	<0.01
Chronic pain severity	0.188 (0.032, 0.343)	0.018
Type of surgery		
Gynecologic surgery	-0.823 (-1.441, -0.206)	0.090
Orthopedic surgery	0.600 (-0.554, 1.754)	0.308
Pain intensity		
Worst pain	0.240 (0.117, 0.363)	<0.01
Current pain	0.205 (0.112, 0.298)	<0.01

Abbreviations: CI, confidence interval; ASA PS-I, American society of Anesthesiologists physical status classification I.

*Variables tested that did not predict interference with movement included age, ethnic group, type of surgery and time spent in severe pain. Variables that did not predicted activities in bed and sleep included age, gender, educational status, marital status, physical status, ethnic group, religion, type of surgery and anesthesia, chronic pain severity and time spent in severe pain. Variables tested but not predicted interference with breathing and coughing included sex, religion, marital status, and ethnic background. Variables tested that did not predicted anxiousness and helplessness are age, gender, educational status, and least pain intensity.

†For risk factors with only main effects, the coefficient indicates mean difference from the reference group (e.g., the reference group for marital status is married, for educational status is literate, for type of anesthesia is general anesthesia, for type of surgery is general surgery, for physical status is ASA PS I, for religion is orthodox, for ethnicity is Amhara). Time is treated as continuous variable because of linearity of outcome variable over time.

#### B. Interference with activities in bed

The mean (SD) NRS scores for pain interference with activities in bed were 5.7 (2.1), 5.0 (1.9), 4.1 (1.9), and 3.0 (2.0) at 6, 12, 24, and 48 hours after surgery, respectively. As final covariates of pain interference with activities in bed, the QIC statistic suggested time since surgery, time in pain, pain intensity (worst, current and time in pain), and perceived pain relief. The worst pain intensity (β = 0.319, 95% CI: 0.225, 0.413), current pain intensity (β = 0.282, 95% CI: 0.174, 0.390), and duration of time patients spent in severe pain (β = 0.021, 95% CI: 0.015, 0.027) significantly predicted the intensity of interference with activities in bed. As time after the surgery elapsed, the intensity of interference decreased (β = -0.021, 95% CI: -0.027, -0.015); this finding was not affected by the amount of relief perceived by the patient (see Table 3).

#### C. Interference with breathing and coughing

Pain interfered with breathing and coughing mildly at 6, 12, 24, and 48 hours after surgery, with mean (SD) NRS scores of 3.0 (2.3), 2.7 (2.1), 2.3 (2.0), and 1.6 (1.8), respectively. Less pain interference with coughing and breathing was reported by those who underwent spinal anesthesia and orthopedic procedures (β = -1.222, 95% CI: -1.879, -0.565 and β = -1.235, 95% CI: -2.135, -0.335, respectively). Patients with chronic pain reported greater interference with breathing and coughing (β = 0.253, 95% CI: 0.100, 0.407); this interference with breathing decreased with increasing perceived pain relief (β = -0.020, 95% CI -0.037, -0.004) and time after surgery (β = -0.015, 95% CI: -0.026, -0.004). ASA PS 2 patients reported greater interference of pain with breathing and coughing (β = 0.671, 95% CI: 0.022, 1.321) than ASA PS 1 patients. Sociodemographic variables, such as sex, religion, marital status, and ethnic background, showed no effect (see Table 3).

#### D. Interference with sleep

The mean NRS (SD) scores of pain interference with sleep at 6, 12, 24, and 48 hours after surgery were 3.4 (2.2), 3.0 (2.0), 2.4 (1.9), and 1.6 (1.7), respectively. The worst pain intensity (β = 0.352, 95% CI: 0.211, 0.493), current pain intensity (β = 0.302, 95% CI: 0.182, 0.421), time in severe pain (β = 0.021, 95% CI: 0.011, 0.030), and relief received (β = -0.022, 95 CI: -0.033, -0.011) were strongly associated with pain interference with sleep, but age, duration of surgery, preoperative pain intensity, and least pain showed no effect (see Table 3).

#### E. Interference with emotions

At 6, 12, 24, and 48 hours after surgery, the mean (SD) NRS scores for feelings of anxiousness as a result of pain were 2.2 (2.1), 1.9 (1.9), 1.5 (1.6), and 1 (1.4) and the mean (SD) scores for pain causing a feeling of helplessness were 1.5 (1.6), 1.3 (1.6), 0.9 (1.3), and 0.7 (1.3), respectively. Single participants had less pain interference with anxiousness (β = -0.957, 95% CI: -1.649, -0.265) and feelings of helplessness (β = -0.727, 95% CI: -1.408, -0.046) than married participants. Muslims scored higher on pain causing helplessness than Orthodox Christians (β = 0.418, 95% CI: (0.003, 0.833). Gynecologic surgery patients had less anxiousness (β = -1.002, 95% CI: -1.685, -0.319) and helplessness (β = -0.823, 95% CI: -1.441, -0.206) than general surgery patients. Higher chronic pain NRS scores were associated with increased anxiousness (β = 0.179, 95% CI: 0.005, 0.352) and helplessness (β = 0.188, 95% CI: 0.032, 0.343). A similar trend was noted for the worst pain intensity: The more intense the worst pain, the higher the rating of anxiousness (β = 0.308, 95% CI: 0.179, 0.437) and helplessness (β = 0.240, 95% CI: 0.117, 0.363). Current pain intensity affected pain causing helplessness (β = 0.205, 95% CI: 0.112, 0.298), but not anxiousness. Age, sex, level of education, and ethnic background showed no effects (see Table 3).

### Patient satisfaction

The mean (SD) patient satisfaction as indicated by the NRS scores was 6.8 (1.6) at 6 hours, 7.2 (1.4) at 12 hours, 7.6 (1.3), and 7.9 (1.4) at 48 hours after surgery. Ethnic background, pain interference, and perception of care were associated with patients’ ratings of satisfaction. The only pain intensity variable found to have any correlation with patients’ ratings of satisfaction was the time spent in severe pain (β = -0.011, 95% CI: -0.020, -0.001). An increase in pain interference with activities in bed decreased patient satisfaction (β = 0.097, 95% CI: -0.392, -0.012). Pain interference with sleep was associated positively with satisfaction (β = 0.258, 95% CI: 0.049, 0.468). The degree to which a patient felt relief from pain was also associated with the level of satisfaction (β = 0.031, 95% CI: 0.012, 0.051). Time since surgery, sex, marital status, religion, type of anesthesia, preoperative chronic pain, type of surgery, and patients’ worst, least, and current pain intensity were not significantly associated with satisfaction (see Table 4).

**Table 4:** Multivariate model predicting patient satisfaction over time*.

Predictor	Coefficient (95% CI)^†^	P Value
Ethnic group		
Oromo	0.512 (0.023, 1.002)	0.040
Others‡	0.652 (-0.003, 1.306)	0.050
Time in pain	-0.011 (-0.020, -0.001)	0.028
Pain interference with function		
Activities in bed	-0.202 (-0.392, -0.012)	0.037
Sleeping	0.258 (0.049, 0.468)	0.016
Relief received	0.031 (0.012, 0.051)	0.002

Abbreviations: CI, confidence interval.

*Variables tested that did not predict satisfaction included age, gender, marital status, educational status, chronic pain severity, type of anesthesia, type of surgery, American Society of Anesthesiologists physical status classification, time since surgery, worst pain intensity, least pain intensity, current pain intensity.

†For risk factors with only main effects, the coefficient indicates mean difference from the reference group (eg, the reference group for Ethnic group is Amhara).

‡Tigre, Wolayta, Gurage, Kafa, Silte.

### Subgroup analysis

**Fig 3:**
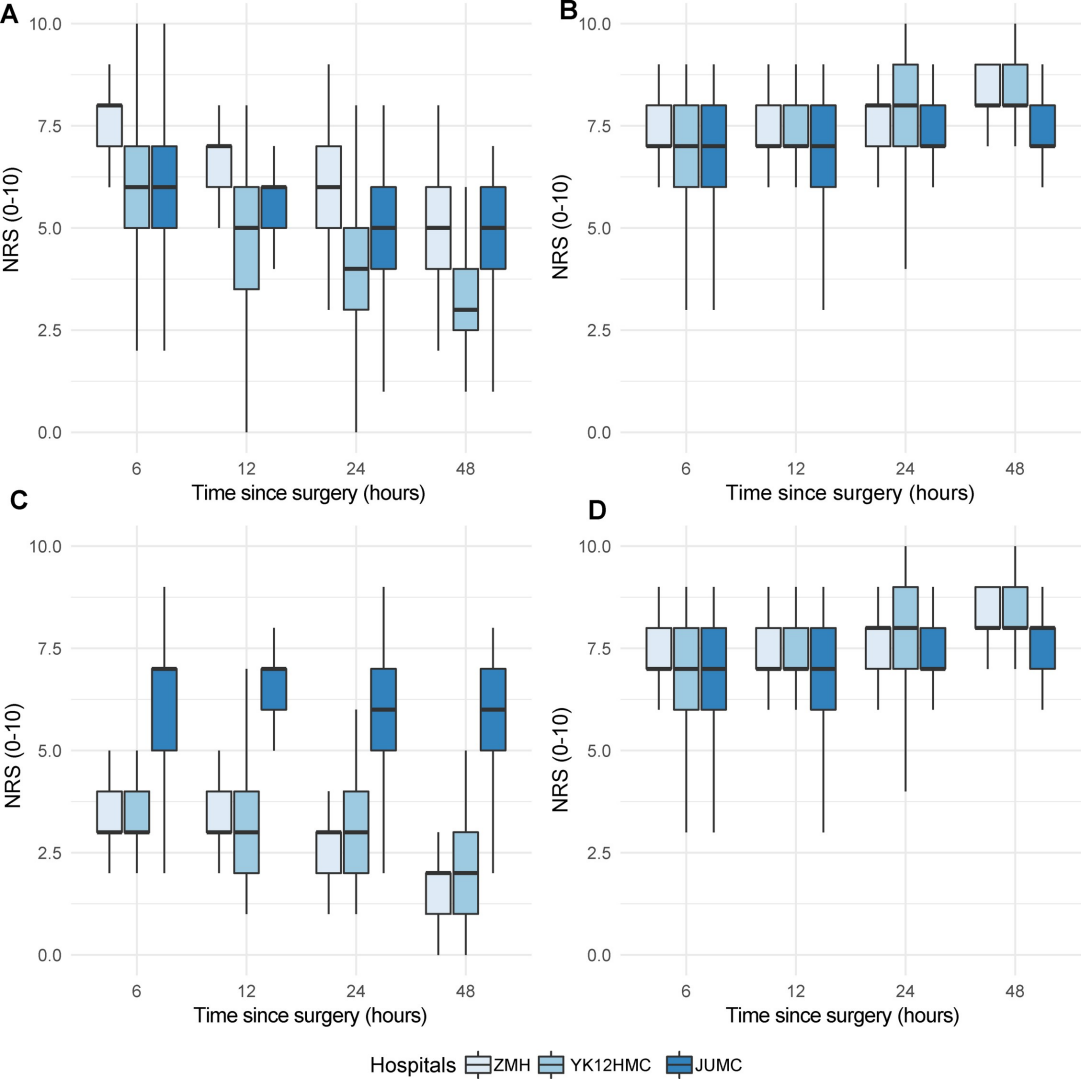
Numeric rating scale (NRS) scores of patients among hospitals. (A) worst pain intensity. (B) satisfaction. (C) time spent in severe pain and (D) pain relief received.

To compare the hospitals, we carried out an analysis of a subgroup of 306 patients who underwent general and gynecologic surgery. Orthopedic patients were excluded because one of the participating hospitals (ZMH) do not contribute to the population. Significant differences between the three hospitals were observed in worst pain intensity score at 24 and 48 hours (β = 0.831, 95% CI: 0.265, 1.40 and β = 1.7, 95% CI: 1.130, 2.30, respectively). At 48 hours patients from YK12HMC reported significantly lower satisfaction level (β = -1.184, 95%CI: -1.839, -0.529) and a higher percentage of time spent in severe pain (β = 1.379, 95% CI: 0.799, 1.959). Compared with JUMC, patients at ZMH had reported higher perceived pain relief at 12, 24 and 48 hours postoperatively (β = 1.035, 95% CI: 0.155, 1.914, β = 1.347, 95% CI: 0.468, 2.226 and β = 0.090, 95% CI: 0.211, 1.969, respectively). (Fig 3).

## Discussion

We provide substantial evidence that postoperative pain management at the three study centers, which were more or less representative for Ethiopia, was insufficient and that pain impaired patients’ physical functioning and emotional well-being during the postoperative period. Most of the analgesics were prescribed by the surgical residents. This seems to have an important implication for the destiny of pain management in the setting. For instance, in battling the opioid crisis, developed world have already identified that agents of change should be the surgical resident as larger amount of opioid were prescribed by them [22]. In the same manner Ethiopia might be benefited if started early to target these group of professionals (surgical resident) to halt the possible tramadol crisis [23]. A high prevalence of moderate to severe postoperative pain was observed. Such a high prevalence might be comparable to studies conducted the early 2000s; for example, a study in the USA reported a prevalence of up to 86% [24]. However, the prevalence found in our study is unacceptably high compared with recent studies from both developed [25] and developing countries [9], which reported prevalences of 34% and 62%, respectively. Even two days after surgery (48 h), 40% of patients were in moderate to severe pain, which is still higher than in other settings in Africa [26].

The observed magnitude of pain could originate from interactions of heterogeneous but interrelated factors. First, the poor knowledge of health care providers (HCPs) about pain and their attitude towards it play a role; established evidence already exists to support this argument [27]. A nationwide study conducted in Ethiopia confirmed this finding: according to this report, the majority of HCPs were not competent in either treating or assessing pain [28]. Second, a lack of organizational commitment, resources, and supervision could also inflame the high prevalence of pain in hospitalized patients [5]. Third, some authors argue that high pain scores are a consequence of inadequate doses of analgesics [29]. The high frequency of negative scores we observed on the pain management index may be relevant in this context. Tramadol alone was mainly used (92.9%), and diclofenac alone occasionally (7%), which is again contrary to international recommendations [30].

This study also uncovered a mismatch between patients’ pain intensity and the strength of analgesics prescribed. The calculated PMI indicated that 58.4% of participants received sub-optimal pain treatment at 6 hours after surgery; a study from China reported similar results [16]. However, caution must be exercised when interpreting the results of pain management index. Previous studies have already identified that though pain intensity is expected to decrease as the PMI score increases this is not always true [31]. For example, in this study, only 2.5% of patients were inadequately treated at 24 hours, despite the fact that about 40% needed more analgesics than prescribed at this particular time. This could be because of several reasons; for one thing, this index cannot differentiate between an analgesic that is prescribed and administered [32]; as the index was originally developed to measure physicians’ reaction to patients’ pain [33]. Secondly, Sakakibara, N., et al., have identified that those with –1 score (therefore bad pain control, according to PMI), have scored more pain interference than those with PMI score 0 (good pain control). The authors recommended that when the aim is to identify definitively inadequate care, PMI scores of − 2 and − 3 should be considered, and not scores of − 1 [31]. In addition in spite of receiving strong opioids, previous reports have shown that approximately 85% of patients reported uncontrolled postoperative pain [32, 34]. The bottom line is that PMI is very optimistic in measuring the quality of pain management and a careful interpretation is mandatory [32].

None of the patients in this study received information about pain treatment options. This result is not surprising because there was no supervision of the HCPs’ pain management practices or acute pain services in the country [28]. In fact, a study conducted in Iceland reported that 70% of patients did not receive information on pain treatment options [35]. Nowadays, it is strongly recommended to provide preoperative information to patients to improve acute postoperative pain [36]. As those patients’ who received a preoperative information have lower preoperative anxiety and therefore a lower postoperative pain intensity [37]. This partly explains why no significant differences were observed for most of the postoperative periods among participating hospitals. Patients at YK12MC hospital reported higher pain intensity at 24 and 48 hours, including less satisfaction at 48 hours compared to the JUMC. As the analysis were adjusted for both clinical and demographic patient variables this cannot be explained by differences in mean population characteristics. Rather this could be due to differences between hospitals related to the HCPs’ level of training, communication gap among HCPs, empathy towards patients [38] or even differences related to culture [39].

We noted a link between pain intensity and interference with physical function. However, pain impaired patients’ activities in bed more than their physical movements when out of bed. This might be because patients will not move around when out of bed unless the pain drops to a tolerable level. Furthermore, mobility is affected by the nature of the surgical procedures: early in the postoperative period, orthopedic patients resumed movement somewhat later than non-orthopedic patients. This finding is similar to previous studies, which reported a positive correlation between pain intensity and interference [40].

In line with other investigations, preoperative pain contributed to higher postoperative pain ratings [41–43]. Because the brain is no longer considered to be a fixed organ, the effect of chronic preoperative pain on postoperative pain intensity can be interpreted according to the principles of neural plasticity [44, 45]. In a study that used a transcutaneous electric sensation in surgical patients, researchers reported preoperative back pain to be associated with central neuroplasticity [45]. Although analysis revealed statistically significant association between age and worst pain intensity (p = 0.014), it is barely clinically significant result given the size of the effect estimate (β = -0.018) [46]. The absence of finding of a relationship between age and pain intensity is not new [47–49]. However, researchers have observed less pain-related caudate and putamen activity of the brain in healthy older adults than in younger adults [50]. Nevertheless, conclusive evidence is needed to determine whether older individuals underreport pain or have lower pain sensitivity [43]. In keeping with pain intensity, our results indicate that gender is not relevant. A very recent study affirmed this by showing how age and preoperative pain could be confounders, rather than actually being associated [7, 51]. In a recently published review, gender differences in pain were found to be inconsistent after orthopedic and abdominal procedures and absent after oral surgery [51].

The influence of ethnicity [52], religion and spirituality [53] on postoperative pain intensity has been studied. Especially researchers who investigated the impact of spirituality on pain have recommended prayer and meditation interventions as non-pharmacological alternatives to treat pain [53]. In our study, neither religion nor ethnicity was associated with the patient’s worst pain intensity. Nonetheless, the data from our study could neither confirm nor deny this finding. Thus, a study is needed in a larger, nationwide cohort to explore to what extent these factors play a role. Little is available in the literature regarding the impact of literacy status on the level of postoperative pain. A study from Greece found out that those with the junior level of educational status experienced more intense pain compared with patients with a higher educational status [54]. The authors concluded that the low educational status is associated with poor understanding of preoperative information, which in turn might cause anxiety, depression, and suboptimal use of analgesia [54]. However, in our study, this could not explain why illiterate patients reported higher pain intensity; as no patients reported receiving preoperative information at all. Similarly, Whelan et al., after analyzing 5584 hospitalized patients found that patients with higher levels of education reported more significant pain and were less satisfied with their pain management [55]. These conflicting results should be well investigated in the future. Our finding shows that single patients reported more intense pain than their married counterparts. Similarly, Schade et al., demonstrated that support from the patient’s spouse was an independent predictor of long-term postoperative pain relief [56]. In another study of 56 male patients who underwent coronary bypass surgery, married patients recovered more quickly and consumed fewer analgesics than their unmarried counterparts [57]. However, following spinal surgery Adogwa, et al. reported no significant advantage of marriage (social support) for both short and long-term clinical outcome [58].

Although it is puzzling, despite high levels of pain intensity patients in this study reported a higher level of satisfaction. This has been termed the “severe pain-high satisfaction paradox” [59], and seems to be a common finding [60, 61]. This paradox has been interpreted in many ways, and HCPs’ caring attitudes towards patients was one possible explanation, i.e. HCPs’ compassionate care might diminish the patients’ pain experience and improve their satisfaction [60]. However, our unpublished qualitative study seems to indicate quite the opposite because our patients criticized their respective HCP for a lack of empathy in pain treatment. Also, postoperative pain might be unavoidable in patients’ minds, and they may perceive it as normal; this, in turn, might affect patient satisfaction [61]. Another study found that neither age nor gender affected patients’ ratings of satisfaction [55]. Our results support previous reports of a negative correlation between satisfaction and time spent in severe pain and a positive correlation with the perceived relief received [62]. Some might wonder why we observed a positive association between ratings of satisfaction and pain interference with sleep in our study. First, the overall level of pain interference with sleep was quite low in our sample, so it would not have been enough to negatively affect a larger number of patients’ reports of satisfaction. Second, although not directly associated with pain interference with sleep, such unexpected findings are not uncommon when it comes to patient satisfaction in postoperative pain management. For example, a positive correlation between satisfaction and adverse events was observed previously [62]. Moreover, some people believe that the measure of satisfaction is not a reliable indicator of the quality of postoperative pain treatment and should not be used [63]. Nevertheless, future investigations on the relationship between pain interference and satisfaction require populations with higher ratings of pain interference with sleep. Last, previous investigations have explored the relationship between satisfaction and background ethnicity [40]. Although our results indicate an association, given our sample size we would not go so far as to draw a conclusion. Our study found no indication for an association between the patient rating of satisfaction and worst, current, or least pain intensity; this result is similar to previous investigations [60, 64].

As to the strengths of the study, to our best knowledge, this was the first prospective longitudinal study using data from three tertiary hospitals to evaluate the quality of pain management in elective gynecologic, surgical, and orthopedic patients in Ethiopia. The study also attempted to apply modern and advanced methods of statistical analysis, which are recommended by experts in the field [65]. We studied a relatively representative population by including three major teaching and referral hospitals in Ethiopia. Hence, our findings contribute to the growing database on the experience of postoperative pain treatment in low-resource countries, where a lack of research on the topic is one barrier to improving the quality of pain treatment. Future studies should design and implement an intervention to lower the already identified magnitude of patients suffering under treated postoperative pain. To this end we are implementing an interdisciplinary pain education (including patients), to improve the quality of postoperative pain management.

An inherent limitation of an observational study design is that it does not allow us to draw conclusions about causal inferences or temporality. We cannot entirely rule out the possibility of other confounders or other explanatory models when determining the association between chronic pain and postoperative pain intensity or between age and postoperative pain intensity. In addition, we assessed only a limited set of variables that could explain their relationships. The identified risk factors and predictors are not the only models that could be used to examine the link between clinical and sociodemographic characteristics and postoperative pain intensity, and alternative models (e.g., adding preoperative anxiety or intraoperative use of analgesics) could be used to explore other relationships. Unlike most surveys we had a full response rate which contributes positively to the study by minimizing the nonresponse bias. Since, in most surveys about 60% response rate is reported to be satisfactory the power of this study is sufficient for determining a strong association between the predictors and the outcome measures [66, 67]. However, in terms of response bias, patients may give response to questions by reporting what they believe should be reported rather than what they actually feel. In addition, the positive or negative encounter they had that day with HCP happen to be caring for them might have affected their response [67].

## Conclusions

This study suggests that postoperative pain is not well managed at hospitals in Ethiopia and that there is an unacceptably high prevalence of moderate to severe postoperative pain. It also provides evidence indicating a severe interference of pain with patients’ functional activities in bed, which could result in several complications. It is important to aim to improve the quality of postoperative pain management. Additionally, this report confirmed that patients are satisfied with the postoperative care provided to them, despite having higher pain intensity scores. In limited resource settings, attention needs to be paid to implementing interventions that reduce the amount of pain and aim to deliver high-quality postoperative pain management.
